# The impact of comorbidities and COVID-19 on the evolution of community onset sepsis

**DOI:** 10.1038/s41598-023-37709-6

**Published:** 2023-06-30

**Authors:** Giovanna Colantuono de Araújo, Andrea Pardini, Camila Lima

**Affiliations:** 1 Nursing Course, Israeli Faculty of Health Sciences Albert Einstein, São Paulo, São Paulo Brazil; 2 Present Address: Medical Surgical Nursing Department, Nursing School of the University of São Paulo, 419 Av. Doutor Enéas Carvalho de Aguiar, Third Floor, Cerqueira César, São Paulo, 05403-000 Brazil

**Keywords:** Risk factors, Comorbidities, Infection

## Abstract

Sepsis is a disease with high mortality and morbidity despite advances in diagnostic procedures and therapeutic strategies. The aim of this study was to evaluate the profile and outcomes of community-onset sepsis. This retrospective, multicenter study included five 24-h health care units and was conducted from January 2018 to December 2021. Patients were diagnosed with sepsis or septic shock according to the Sepsis 3.0 criterion. A total of 2630 patients diagnosed as having sepsis (68.4%, 1800) or septic shock (31.6%, 830) in the 24-h health care unit were included; 43.76% of the patients were admitted to the intensive care unit, 12.2% died, 4.1% had sepsis and 30% had septic shock. The comorbidities that were independent predictors of septic shock were chronic kidney disease on dialysis (CKD-d), bone marrow transplantation and neoplasia. CKD and neoplasia were also independent predictors of mortality, with ORs of 2.00 (CI 1.10–3.68) p = 0.023 and 1.74 (CI 1.319–2.298) p =  < 0.0001, respectively. Mortality according to the focus of primary infection was as follows: pulmonary 40.1%; COVID-19 35.7%; abdominal 8.1% and urinary 6.2%. Mortality due to the COVID-19 outbreak had an OR of 4.94 (CI 3.08–8.13) p ≤ 0.0001. Even though community-onset sepsis can be potentially fatal, this study revealed that some comorbidities lead to an increased risk of septic shock (d-CKD and neoplasia) and mortality. COVID-19 infection as the primary focus was an independent predictor of mortality in patients with sepsis when compared to other foci.

## Introduction

Sepsis is considered a major cause of health loss; an estimated 48·9 million incident cases of sepsis were recorded worldwide, and 11·0 million sepsis-related deaths were reported, representing 19·7% of all global deaths^1^.

Approximately 15% of patients with sepsis experience septic shock, which accounts for approximately 10% of all intensive care unit (ICU) admissions and has a mortality rate of approximately 50%^1^.

Some concepts of sepsis definition and management have evolved in recent decades, and the last international consensus occurred in 2016, known as Sepsis-3.0^2^. Sepsis is an extreme response to infection caused by a dysregulated immune response to bacterial, viral, fungal and protozoan infection and potentially causing major organ dysfunction^2^ The diagnosis of viral etiology sepsis, particularly among patients with COVID-19, is rare^3–5^, indicating reduced adherence to the sepsis protocol. The meta-analysis including more than 37,000 patients showed that the incidence of viral sepsis due to COVID-19 was 17.7% (95% CI 12.9–23.6) in the ward and 77.9% in the ICU (95% CI 75.9–79.8)^6^.

There are few studies reporting on community-onset sepsis, such as its incidence in 24-h health care units, risk factors (comorbidities) and focus of primary infection in individuals with nonhospital sepsis. The aim of the present study was to evaluate the prevalence of sepsis in the community in pre- and pandemic periods and its clinical outcomes.

## Results

### Cohort characteristics, comorbidities and outcomes

A total of 911,549 were treated at the 24-h health care units, 2630 (0.29%) patients diagnosed as having sepsis (68.4%, 1800) and septic shock (31.6%, 830) in the 24-h health unit were included. The characteristics of the cohort are presented in Table 1. The mean age was 73.55 years (SD, 17.55) and individuals who were age 72.39 years (SD, 16.89) had a higher incidence of septic shock than those age 74.03 years (SD, 17.83). A total of 1557 (59.2%) of the sepsis cohort were males (p 0.001). Previous hospitalizations in the last 30 days were present in 282 (10.7%) of patients and they were not excluded from the analyses.

**Table 1:** Cohort characteristics, comorbidities and outcomes.

	Total	Sepsis	Septic shock	Q
Age	73.52 DP (17.55)	74.03 DP (17.83)	72.39 DP (16.89)	0.001
Sex	1557 (59.2%)	1058 (58.8%)	498 (60%)	0.605
Comorbidities				
Neoplasm	442 (16.8%)	278 (62.9%)	164 (37.1%)	0.004
Marrow_Tx	12 (5%)	4 (33.3%)	8 (66.7%)	0.013
CKD_HD	65 (2.5%)	28 (43.1%)	37 (56.9%)	< 0.0001
CKD_No_HD	153 (5.8%)	97 (63.4%)	56 (36.6%)	0.1
Tx_Kidney	17 (0.6%)	11 (64.7%)	6 (35.3%)	0.46
CHF	274 (10.4%)	197 (71.9%)	77 (28.1%)	0.107
AMI_angina_ICO	127 (4.8%)	87 (68.5%)	40 (31.5%)	0.535
Heart disease	563 (21.4%)	379 (67.3%)	184 (32.7%)	0.28
SAH	1112 (42.3%)	749 (67.4%)	362 (32.6%)	0.184
COPD	257 (9.8%)	182 (70.8%)	75 (29.2%)	0.212
Diabetes mellitus	713 (27.1%)	478 (67%)	235 (33%)	0.19
CIRRHOSIS	15 (0.6%)	8 (53.3%)	7 (46.7%)	0.162
Liver_tx	9 (0.3%)	7 (77.8%)	2 (22.2%)	0.42
HIV	22 (0.8%)	17 (77.3%)	5 (22.7%)	0.258
Brain stroke	200 (7.6%)	136 (68.3%)	63 (31.7%)	0.981
Alzheimer’s dementia	326 (12.4%)	229 (70.2%)	97 (29.8%)	0.244
Charlson index	1.95 (2.14)	1.84 (2.06)	2.16 (2.29)	< 0.0001
Prior hospitalization 30 days	282 (10.7%)	181 (10.1%)	101 (12.2%)	0,107
Outcomes				
SAPS3	52.10 DP (10.15)	50.29 DP (8.82)	55.86 DP (11.62)	< 0.0001
SOFA	3.36 DP (2.14)	2.93 DP (1.47)	4.29 DP (2.29)	< 0.0001
Time in ICU	5.59 DP (13.07)	4.33 DP (10.35)	8.31 DP (17.27)	< 0.0001
Length of hospital stay	17.48 DP (24.71)	13.37 DP (18.10)	26.40 DP (33.31)	< 0.0001
Need for RRT	263 (10%)	62 (23.6%)	201 (76.4%)	< 0.0001
Mortality	322 (12.2%)	73 (4.1%)	249 (30%)	< 0.0001

Data are expressed as n (%), mean ± SD, median and percentile (25–75) according to their distribution.

*tx* transplant, *CKD* chronic kidney disease, *HD* hemodialysis, *CHF* congestive heart failure, *AMI* acute myocardial infarction, *ICO* coronary insufficiency, *SAH* systemic arterial hypertension, *COPD* chronic obstructive pulmonary disease, *HIV* human immunodeficiency virus, *ICU* intensive unit care, *SAPS* Simplified Acute Physiology Score, *SOFA* Sequential Organ Failure Assessment, *RRT* renal replacement therapy.

The comorbidities were systemic arterial hypertension (1112, 42.3%), diabetes mellitus (713, 27.1%), heart disease (563, 21.4%), neoplasms (442, 16.8%), dementia (326, 12.4%), heart failure (274, 10.4%), chronic obstructive pulmonary disease (9.8%), stroke (7.6%), chronic kidney disease (CKD) not on dialysis (5.8%), acute infarction myocardial infarction (4.8%), CKD on dialysis (5.8%), human immunodeficiency virus (HIV) (0.8%), kidney transplant (tx) (0.6%), cirrhosis (0.6%), bone marrow tx (0.5%) and liver tx (0.3%). The Charlson index was 1.95 (SD, 2.14) and was higher in patients with septic shock (2.16 SD (2.29) than in those with sepsis (1.84 (2.06), p < 0.0001).

Ninety-seven (3.69%) patients required hospitalization, 1382 (52.55%) were admitted in the semi-intensive care unit, and 1151 (43.76%) were admitted in the intensive care unit (ICU). The length of stay in the ICU was 5.59 days (SD, 13.07), and the hospital stay was 17.48 days (SD, 24.71). Two hundred sixty-three patients (10%) needed renal replacement therapy, and the lengths of ICU and hospital stays and the need for RRT were significantly greater in patients with septic shock (p < 0.0001).

**Figure 1:**
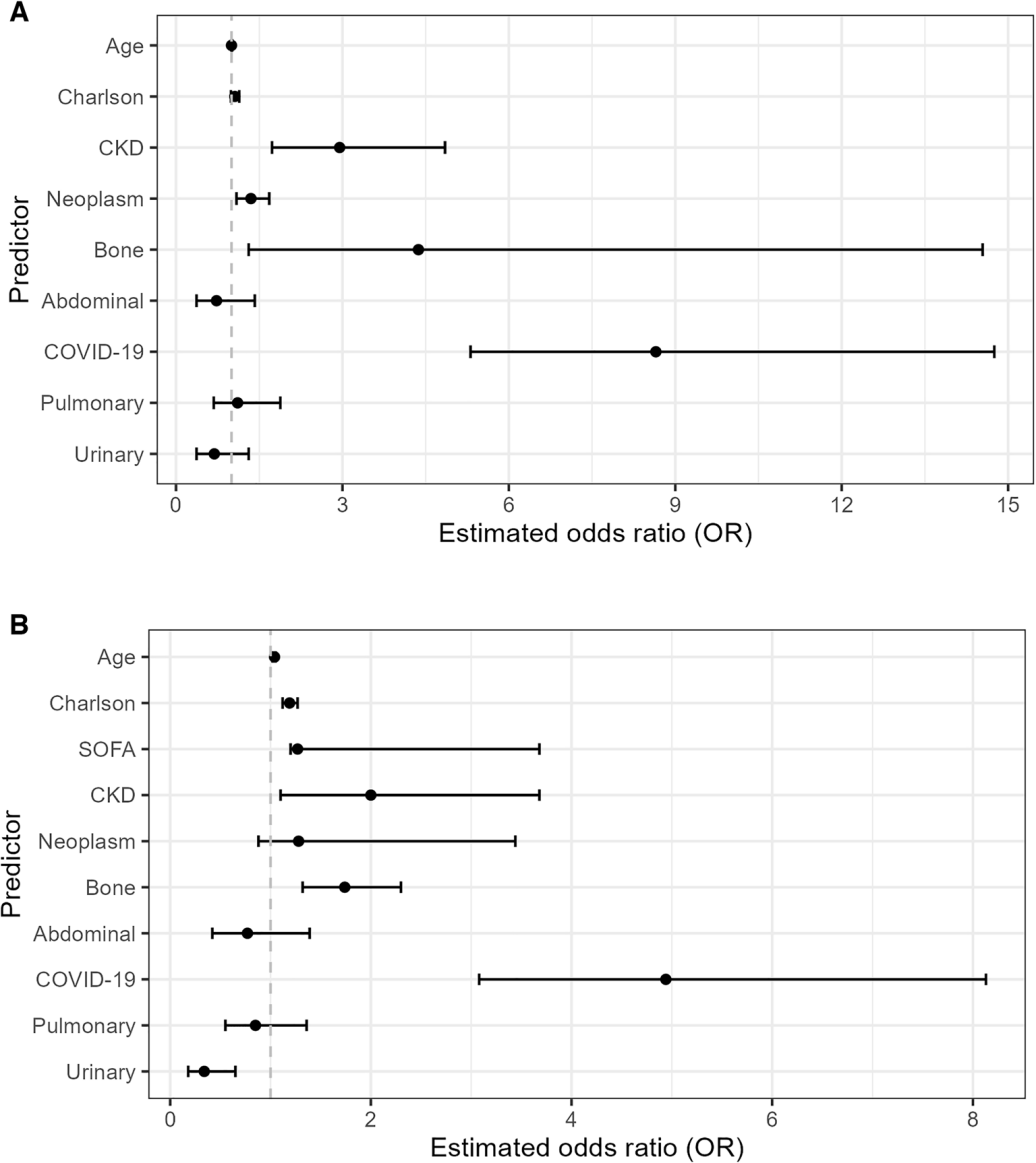
Variables included in the logistic regression of progression to septic shock (**A**) and mortality (**B**). *CKD* chronic kidney disease, *SOFA* Sequential Organ Failure Assessment.

The mortality rate for all patients was 12.2%, with a 4.1% rate in sepsis patients and a 30% rate in septic shock patients (p < 0.0001). The comorbidities most associated with septic shock were CKD on dialysis 56.9%—(OR 2.949, CI 1.792–4.853, p ≤ 0.0001), bone marrow transplant 66.7% (OR 4.365, CI 1.31–14.54, p = 0.013), and neoplasms 37.1% (OR 1.346, CI 1.09–1.68, p = 0.004) (Table 2, Fig. 1). The comorbidity variables that were found to be independent predictors of mortality were CKD on dialysis (OR 2.00, CI 1.10–3. 68, p = 0.023), neoplasia (OR 1.74, CI 1.319–2.298, p ≤ 0.0001.

**Table 2:** Odds ratio for septic shock and mortality outcomes.

Parameters:	Septic shock			Mortality		
	Odds ratio	IC	p value	Odds ratio	IC	p value
Age	1.00	(1.00–1.01)	0.365	1.04	(1.02–1.05)	< 0.001
Charlson index	1.06	(0.99–1.14)	0.073	1.19	(1.12–1.27)	< 0.001
SOFA score				1.27	(1.20–1.34)	< 0.001
CKD	2.95	(1.73–4.85)	< 0.0001	2.00	(1.10–3.68)	0.023
Neoplasm	1.35	(1.09–1.68)	0.004	1.28	(0.88–3.44)	0.196
Bone marrow transplantation	4.37	(1.31–14.54)	0.013	1.74	(1.32–2.30)	< 0.0001
Abdominal Focus	0.73	(0.37–1.42)	0.359	0.77	(0.42–1.39)	0.385
COVID-19 focus	8.65	(5.31–14.75)	< 0.001	4.94	(3.08–8.13)	< 0.001
Pulmonary focus	1.11	(0.68–1.88)	0.689	0.85	(0.55–1.36)	0.494
Urinary focus	0.69	(0.37–1.31)	0.252	0.34	(0.178–0.65)	0.001

*CKD* chronic kidney disease, *SOFA* Sequential Organ Failure Assessment.

The most prevalent primary infection foci were as follows: pulmonary sites, 1135 (43.2%); urinary sites, 469 (17.8%); COVID-19 infection, 434 (16.5%), abdominal sites, 316 (12%) and other, 276 (10.5%). Mortality according to the focus of the primary infection was as follows: pulmonary, 40.1%; COVID-19 infection, 35.7%; abdominal 8.1% and urinary 6.2%. In the logistic regression for mortality with respect to focus, pulmonary OR = 0.85 (CI 0.55–1.36, p = 0.49), COVID-19 OR = 4.94 (CI 3.08–8.13, p ≤ 0.0001), abdominal OR = 0.77 (CI 0.42–1.39, p = 0.10); urinary OR = 0.34 (CI 0.18–0.65, p < 0.0001) (Table 2).

The frequency of comorbidities was similar between the pandemic period (2020–2021) and the prepandemic period (2018–2019). Concerning infection focus, an abdominal focus was more frequent in the prepandemic period (173, 14.1%) than in the pandemic period (143, 10.2%, OR: 1.20, CI 1.06–1.36; p = 0.002), and a pulmonary focus was also more frequent in the prepandemic period (661, 53.7%) than in the pandemic period (474, 33.9%, OR: 1.48, CI 1.37–1.60, p < 0.001). There was no difference in the urinary focus between the prepandemic period (223, 18.1%) and the pandemic period (245, 17.5%, OR: 1.02, CI 0.93–1.12, p = 0.36).

## Discussion

There are unknowns regarding community-onset sepsis, including its incidence, risk factors, need for intensive support, progression to septic shock and mortality. In this study, in a cohort of patients who visited a 24-h health care unit, 43.76% of sepsis patients were admitted to the intensive care unit of a hospital and the overall mortality rate was 12.2%, with a 4.1% rate in sepsis patients and a 30% rate in septic shock patients.

Studies show that the mortality rate in patients with sepsis in the hospital setting was 19.2% versus 8.6% in the community setting^7^. In our cohort, although sepsis patients had a mortality rate of 4.1%, septic shock patients had a high mortality rate of 30%, which is close to the 33% mortality rate in patients with hospital-related sepsis^8^ and suggests that early diagnosis and immediate treatment of this pathology outside the hospital setting is crucial.

Sepsis more often progressed to septic shock in slightly younger and was more common in healthy young people than in those with comorbidities (mean age, 58.0 ± 19.8 years vs. 67.0 ± 16.5 years). However, this group required less intensive support as the length of hospital stay was reduced but the short-term mortality rate was higher^9,10^.

There has been even greater uncertainty regarding the incidence and risk factors for community-onset sepsis. A meta-analysis reported the risk factors for sepsis, including older age, diabetes and other medical conditions (e.g., immunosuppression, lung disease and peripheral arterial disease)^11^. Another study found diabetes (15.75%), neoplasia (14.96%) and HIV infection (12.9%) to be common underlying diseases in patients with sepsis^12^.

In our cohort, comorbidities, neoplasia, bone marrow transplantation and dialysis CKD were independent predictors of septic shock, and neoplasia and dialysis CKD were independent predictors of mortality. A common mediator between them and likely associations reflect an underlying abnormal immune status that may predispose individuals to sepsis^13^.

Sepsis disproportionately affects immunocompromised populations, including recipients of allogeneic hematopoietic cell transplants^14^. In immunosuppressed individuals, sepsis has a higherincidence, presents with subtle clinical findings, and progresses more rapidly than in immunocompetent populations^15^. In our study, bone marrow transplantation had an OR of 4.365 (CI 1.31–14.54, p = 0.013) for septic shock.

Individuals with CKD on dialysis require venous or peritoneal access for therapy, which is prone to infections, and approximately 29.8% of these patients develop sepsis, which is a major cause of mortality in this population^16^. The patients with CKD on dialysis in our cohort had an OR of 2.949 (CI 1.792–4.853, p ≤ 0.0001) for septic shock and 2.00 (CI 1.10–3.68, p = 0.023) for mortality.

Cancer-related sepsis was associated with an adjusted absolute increase in hospital mortality, ranging from 2.2 to 15.2% when compared with noncancer-related sepsis^17^. More than 1 in 5 cancer patients with sepsis need hospitalization. In-hospital mortality in patients with cancer-related sepsis is 28%, compared to 20% in noncancer-related sepsis^8^. Our neoplasm study had an OR of 1.346 (CI 1.09–1.68, p = 0.004) for septic shock and 1.74 (CI 1.319–2.298, p ≤ 0.0001) for mortality.

The etiology of sepsis is essential for health promotion strategies and useful for the community and health professionals. The most prevalent foci found in our cohort were urinary (33%), gastrointestinal (18%) and respiratory (18.26%); the mortality rate was higher in pulmonary disease and COVID-19 foci (40.1% and 35.7%, respectively) and lower in abdominal (8.1%) and urinary (6.2%) foci, respectively. Sources in the literature corroborate these findings regarding the prevalence and higher mortality in patients with pulmonary foci and lower mortality in patients with genitourinary foci^18,19^.

Our study evaluated COVID-19 as a focus of infection and not only as a pathogen, as little was known about the pathology at the beginning of the pandemic. Severe COVID-19 infection increases the serum levels of cytokines and chemocytokines that are common in patients with sepsis^6–9^. Because the treatment of viral sepsis is not clear, this pathology has been underreported for years^3–5^. Our cohort showed that the mortality of sepsis unrelated to COVID-19 infection was higher than that of other foci (OR 4.94, CI 3.08–8.13, p ≤ 0.0001) and was the main independent predictor of progression to septic shock (OR 8.65, 5.31–14.75, p < 0.0001).

This study has some limitations. First, the location of this study was in a private health care unit, which may not reflect the reality of health services in general. Second, the study did not describe the treatment of sepsis and other conditions that may have influence the outcome. Third, because COVID-19 is a viral condition, it may have been partially neglected, and the outcome was not associated with the etiology of the pathogen but with the difficulty in managing viral sepsis. Thus, the study periods were pre- and pandemic periods, so the mortality rates could have been linked to the lack of ICU beds and intensive care resources.

## Conclusion

The lack of worldwide health policies for sepsis may have caused the increase in mortality. In the present study, a group of patients diagnosed with sepsis in health care units were followed until discharge or death, and the need for advanced resources, such as beds in the intensive care unit, is urgent. Our results showed a high septic shock mortality rate.

The study revealed that CKD-d and neoplasia were associated with septic shock and mortality and that COVID-19 infection had a higher mortality rate than other infections.

The incidence of viral infection in sepsis patients with COVID-19 and the association between viral sepsis and COVID-19 in terms of mortality rate remain unknown.

## Materials and methods

### Patients

This was a multicenter, retrospective, cross-sectional and quantitative study including five 24-h health care units. The study period was from January 2018 to December 2021, and a total of 2630 patients were eligible for the study, with follow-up until the clinical outcome (discharge/death). The Ethics Committee of the Hospital Israelite Albert Einstein approved the study under protocol number CAAE: 55693222.0.0000.0071. The need for consent was waived and approved by the Ethics Committee of the Hospital Israelite Albert Einstein because the research did not involve more than minimal risk to the data confidentiality participants, it could not be conducted virtually without waivers, and waivers would not negatively affect the rights and well-being of the participants. The electronic data capture system was “Research Electronic Data Capture” (REDCap) hosted on the servers of Hospital Israelite Albert Einstein. All data are available to researchers in a deidentified manner to protect the identity participant and ensure data integrity. The study sites were five 24-h health care units that were located in the state of São Paulo, Brazil, Morumbi, Perdizes, Alphaville, Ibirapuera and Vila Santa Catarina and managed by a private institution Hospital Israelite Albert Einstein located in the state of São Paulo, Brazil. The “24-h health care unit” is a health facility that provides care for patients with mild-to-moderate conditions. There are no hospital beds in these units. The inclusion criteria were as follows: age older than 18 years with diagnosis of sepsis or septic shock. The exclusion criteria were as follows: patients with a Sequential Organ Failure Assessment (SOFA) score lower than 2 points, patients receiving palliative care and patients with previous dysfunctions related to other diagnoses.

All clinical and research activities being reported are consistent with the Principles of the Declaration of Istanbul, with the Declaration of Helsinki and the Strengthening the Reporting of Observational Studies in Epidemiology (STROBE) guidelines for cohort studies.

### Clinical criteria of sepsis and septic shock

In this study, the Third International Consensus on Definitions for Sepsis and Septic Shock (Sepsis-3), published in 2016, was used to identify adults with suspected infection^2^. The laboratory variables, partial pressure of oxygen (PaO_2_), platelet count, creatinine and bilirubin levels, are necessary for the screening and performance of the score. In addition, the patient’s level of consciousness (Glasgow Coma scale score) and mean arterial pressure (MAP) were recorded^2^. In this evaluation, variables are scored 0 to 4, and patients with 2 or more SOFA points in the presence of an infection is diagnosed with sepsis.

Sepsis is an extreme response to infection caused by a dysregulated immune response to infection, potentially causing fatal organ dysfunction. Septic shock occurs in sepsis patients with nonvolume-refractory hypotension, for which the use of vasopressors is required to maintain a mean arterial pressure (MAP) of 65 mmHg and a serum lactate level equal to or above 18 mg/dL (2 mmol/L)^2^.

### Statistical analysis

The extraction of variables was performed using the database of the REDCap utility. The focus of infection, mortality rate and outcomes such as the incidence of new comorbidities were evaluated.

Categorical variables are expressed as numbers (%), and continuous variables are expressed as the means and standard deviations, whereas nonparametric variables are expressed as medians and 25th–75th percentiles. The Gaussian distribution was determined using the Shapiro‒Wilk test. For the correlation (analysis) of the continuous variable with a normal distribution, the t test or Pearson's test was used, and for continuous variables with a nonnormal distribution, the Mann‒Whitney test (Wilcoxon rank) or the Spearman test was used. Categorical data were compared using the chi-square test or Fisher's exact test. Values of p < 0.05 were considered to be statistically significant. In the logistic regression, we used a model to evaluate the comorbidities and the outcome of septic shock, including the following variables: age, Charlson index score, CKD-d, neoplasia and bone marrow tx; SOFA score was included in the evaluation of comorbidities and mortality. The model to analyze the focus of infection and mortality included age, SOFA, Charlson, abdominal focus, focus, pulmonary, urinary focus and COVID-19 focus. The analyses were performed using the Statistical Package for Social Sciences software (SPSS version 20, Chicago, Illinois).

## Data Availability

The datasets used in this study are not publicly available because of confidentiality, however, it can be made available by the corresponding author Camila Lima upon reasonable request.
